# Commentary on:  Aspartate transaminase to platelet ratio index (APRI): A simple noninvasive index assessing liver fibrosis in patients with chronic liver diseases

**Published:** 2011-05-01

**Authors:** Yilei Mao, Jiuzuo Huang

**Affiliations:** 1 Department of Liver Surgery, Peking Union Medical College Hospital, Chinese Academy of Medical Sciences, Beijing, China

**Keywords:** Chronic hepatitis B, NAFLD, Liver fibrosis, Chronic hepatitis C

Dear Editor,

Hepatitis Monthly has recently published an interesting and practically valuable original report from Yilmaz, et al. [1]. That was a retrospective study investigating the ability of the aspartate transaminase to platelet ratio index (APRI) in diagnosing and predicting the extent of fibrosis in patients with chronic hepatitis B (CHB), chronic hepatitis C (CHC), and non-alcoholic fatty liver disease (NAFLD). This relatively large scale study was conducted on a cohort of 207 CHB patients, 108 CHC patients, and 140 NAFLD patients. The results of that study were apparently reliable and potentially useful with clinical significance. The authors, by comparing with the results from liver biopsy, concluded that APRI has an acceptable accuracy for the assessment of the extent of liver fibrosis in patients with CHC and NAFLD, but not in those with CHB.

Various types of hepatitis, as well as fatty liver disease, have increasingly become an immense problem in both developed and developing countries across the world, which create heavy medical and social burdens. Patients with the said conditions may possess a pathological spectrum of fibrosis, cirrhosis, and ultimately liver cancer. Thus, the information on predicting and staging liver fibrosis is essential for prognostication and decisions on treating patients with chronic liver diseases. Liver biopsy is currently the gold standard for the assessment of liver histology. However, it is invasive and does carry an innegligible risk of complications. Moreover, it could be costly especially to the patients from developing countries. Hence, there is a need to develop an equally-accurate, reliable, noninvasive and inexpensive means to assess liver fibrosis. In the year 2003, Wai, et al. proposed APRI on the basis of routinely available laboratory test results, which were proven to have high sensitivity and specificity in predicting liver fibrosis in patients with CHC. The data were published in Hepatology [2]. Several studies in this field have further revealed the usefulness of APRI in patients with CHC, and a recent systematic review concludes that the major strength of the APRI is the exclusion of significant HCV-related fibrosis [3]. However, the information on the APRI in predicting fibrosis in patients with NAFLD and CHB is still lacking. This study has not only confirmed the results in patients with CHC, but also identified the role of APRI in patients with NAFLD and CHB. Furthermore, most of the previous studies on APRI were conducted in the Western countries, while Yusuf, et al., performed this study in an Asian population. Although other measures such as the Fibro test may undoubtedly achieve a rather similar result, their expense may preclude their actual application in non-wealthy patients. The impact and significant of this study are thus obvious. However, it could be a disadvantage of APRI in the future clinical usage that the upper limits of normal (ULNs) of aspartate transaminase could neither be standardized nor reproducible in different laboratories, which ought to be resolved in further studies.
